# Attitudes of Tunisian psychiatric residents toward sexology: A cross-sectional study

**DOI:** 10.1192/j.eurpsy.2026.12108

**Published:** 2026-07-31

**Authors:** S. Walha, D. Mezri, R. Hosni, A. Ouertani, A. Aissa, R. Jomli

**Affiliations:** Psychiatric department “A”, RAZI Hospital, Mannouba, Tunisia

## Abstract

**Introduction:**

Sexual health has been defined by the WHO as a state of physical, emotional, mental, and social well-being associated with sexuality. It does not consist solely of the absence of disease, dysfunction, or infirmity. To ensure this state of sexual well-being in patients, medical residents, and specifically those in psychiatry, should have the right attitudes to master the physiological and certain pathological aspects of human sexuality.

**Objectives:**

The objective of this study was to assess the attitudes of Tunisian psychiatric residents towards sexuality.

**Methods:**

This was a descriptive and analytical study with cross-sectional data collection conducted on a population composed of a sample of psychiatry residents practicing in Tunisia at all levels: from the first to the fifth year. Our study was based on an online questionnaire and assessed participants’ attitudes of sexuality. It took place from March 1 to April 1, 2025.

**Results:**

A population of 42 young psychiatrists was identified. The average age of these young practitioners was 27.95 years, with a sex ratio of 5. The majority of participants (31%) were fourth-year psychiatry residents. Among the participants, only 21% (n=9) identified themselves primarily as “sexologists” or “sex therapists”." Sexology still accounted for less than 31% of the practitioners’ professional activity, with less than 10% of professional activity in 69% of cases. More than half of the psychiatrists (71%, n=30) only asked questions about sexual dysfunction exceptionally or sometimes during a consultation.

**Conclusions:**

As was the case for other populations surveyed previously in our country, psychiatry residents had a clear lack of certain attitudes regarding sexuality. At the end of this study, we suggest the introduction of some sexology courses as part of the teaching programs at the Faculty of Medicine in Sfax. This will promote the good sexual health of our patients and contribute to better mental balance.

**Disclosure of Interest:**

None Declared

